# The Importance of Prompt Management of Morel-Lavallée Lesions

**DOI:** 10.7759/cureus.24639

**Published:** 2022-05-01

**Authors:** Khizar K Khan, Nareshkumar S Dhaniwala, Sarthak Gupta, Aditya L Kekatpure

**Affiliations:** 1 Orthopedic Surgery, Jawaharlal Nehru Medical College, Datta Meghe Institute of Medical Sciences, Wardha, IND

**Keywords:** pseudocapsule, debridement, subcutaneous necrosis, polytrauma, degloving

## Abstract

The Morel-Lavallée lesion (MLL) is a closed soft-tissue injury that is frequently associated with high-intensity trauma. The thigh, hip, and pelvic regions are the most typically affected regions. It is critical to recognize and treat an MLL as soon as possible because it is often neglected or its identification is delayed because of other distracting injuries in a polytrauma patient. Bacterial colonization of these closed soft-tissue wounds can result in an increased risk of perioperative and postoperative infection. Magnetic resonance imaging has recently been used to define and grade these lesions. To reduce the dangers of these situations, clinical suspicion and on-the-spot identification of these lesions are essential. Here, we report an operated case of fracture shaft femur associated with MLLs and discuss the diagnostic and surgical approaches.

## Introduction

The Morel-Lavallée lesion (MLL) is a kind of degloving injury to traumatized soft tissues that are closed externally. In 1863, French physician Victor-Auguste-François Morel-Lavallée described the lesion for the first time [1]. There is a separation of the hypodermis from the underlying fascia, which can occur when a shearing force is applied to soft tissues [2]. This stimulation disrupts the perforating vascular and lymphatic networks of the soft-tissue envelope, resulting in a characteristic array of hem-lymphatic fluid between tissue layers. The MLL has the potential to significantly influence the treatment of orthopedic injuries. There is a risk of misdiagnosing such lesions in patients with polytrauma because more evident injuries distract from its presence. Inadequate or delayed diagnosis and care might result in unfavorable outcomes such as pseudocyst development, infection, and cosmetic deformity.

The MLL can either present acutely or can occur days after the injury. Several factors determine its presentation. The clinical diagnosis of MLL is often determined by the rate and degree of hemolymphatic accumulation inside the cavity and the body habitus of the patient. The affected areas can display ecchymosis, swelling of the soft tissue, and fluctuation or hypermobility of the skin. Because the superficial skin discoloration may be delayed for many days, initially, the diagnosis can go unidentified. The affected area can become painful and firm with time, which indicates the formation of capsules. Chronic lesions can imitate other diseases of soft tissue including neoplasms. Due to inappropriate management, the late evolution of the lesion may result in soft-tissue envelope infections or necrosis [2]. Ideally, the diagnosis of an MLL is achieved by physical examination of the patient, but advanced imaging modalities can be used to provide additional details. A computed tomography (CT) scan of the affected area is usually performed, particularly when there is acetabular or pelvic injury. Magnetic resonance imaging (MRI) can be used to identify six distinct patterns of lesions. The edges of lesions with MRI images are used to identify each type. The six radiographic characteristics that are used in the classification of each lesion include appearance, the shape of lesions, T1-weighted MRI characteristics, T2-weighted MRI characteristics, and the presence and enhancement of a capsule and lesion.

The patient reported here presented with swelling and blackish discoloration of the skin on the lateral part of the right distal thigh and the proximal aspect of the right leg with ipsilateral operated fracture of distal one-third shaft femur with retrograde interlock nail in situ. Although the site is not typical of MLL, it occurs in the distal thigh and knee region when associated with high-energy Injuries. Such a combination of injuries has not been previously described. Multiple surgical interventions were done resulting in dramatic improvement.

## Case presentation

**Figure 1:**
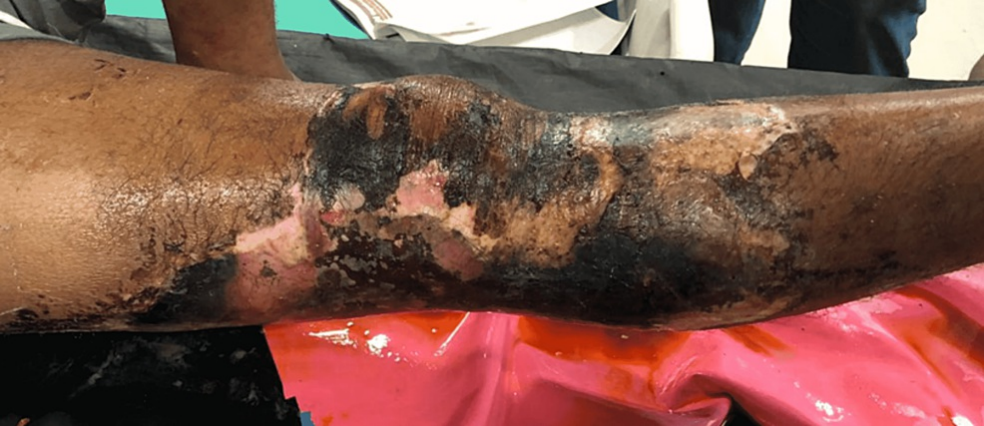
Blackish discoloration of the skin and tense swelling over the thigh, knee, and leg.

A 36-year-old male presented to the Acharya Vinoba Bhave Rural Hospital (AVBRH) casualty department with swelling and blackish discoloration of the skin on the lateral aspect of the right distal thigh and the proximal part of the right leg (Figure 1) as well as discharge from the distal part of the right thigh.

He had met with an accident 18 days back while he was driving a motorcycle and a car hit him from the back. He had a fracture in the right distal one-third shaft femur and bruises on the anterior aspect of the right distal thigh. The fracture was managed with closed reduction and internal fixation with retrograde femur interlock nail in a private hospital.

**Figure 2:**
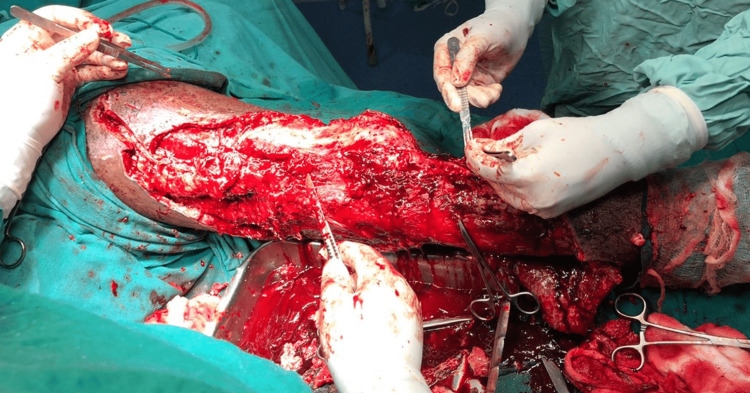
Drainage of the lesion and debridement.

On presentation, he gave a history of the development of blisters over the distal lateral aspect of the right thigh three days after the surgery, followed by pus that started coming from this area 10 days later. His routine investigations showed low hemoglobin, and he was given one unit of packed red cells and two units of whole blood. He was taken up for debridement of the involved area, and 200 mL of purulent fluid lying between the fascia of Vasti muscles and the soft tissue overlying it was drained and sent for culture and sensitivity testing (Figure 2).

**Figure 3:**
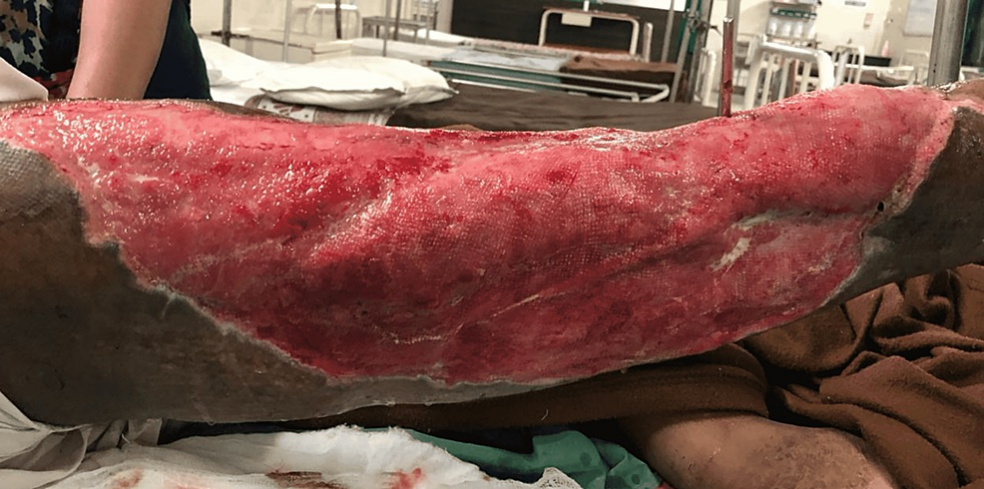
Healthy granulation tissue.

**Figure 4:**
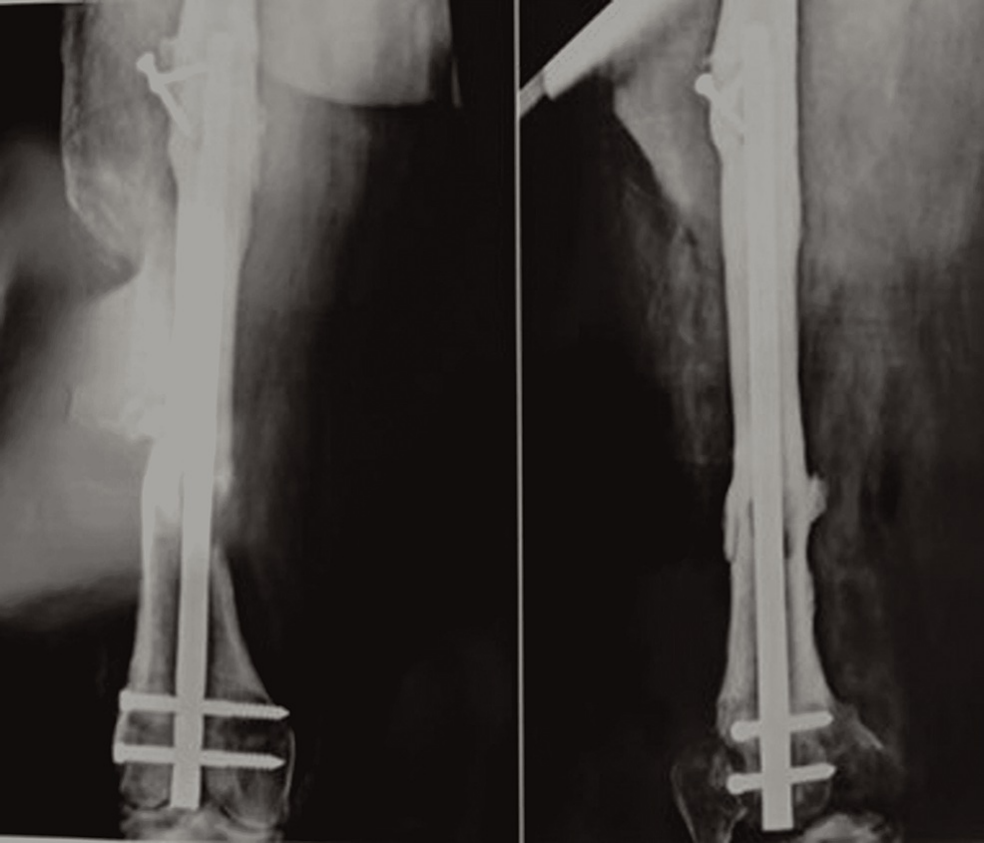
X-ray showing union with the femoral nail in situ.

**Figure 5:**
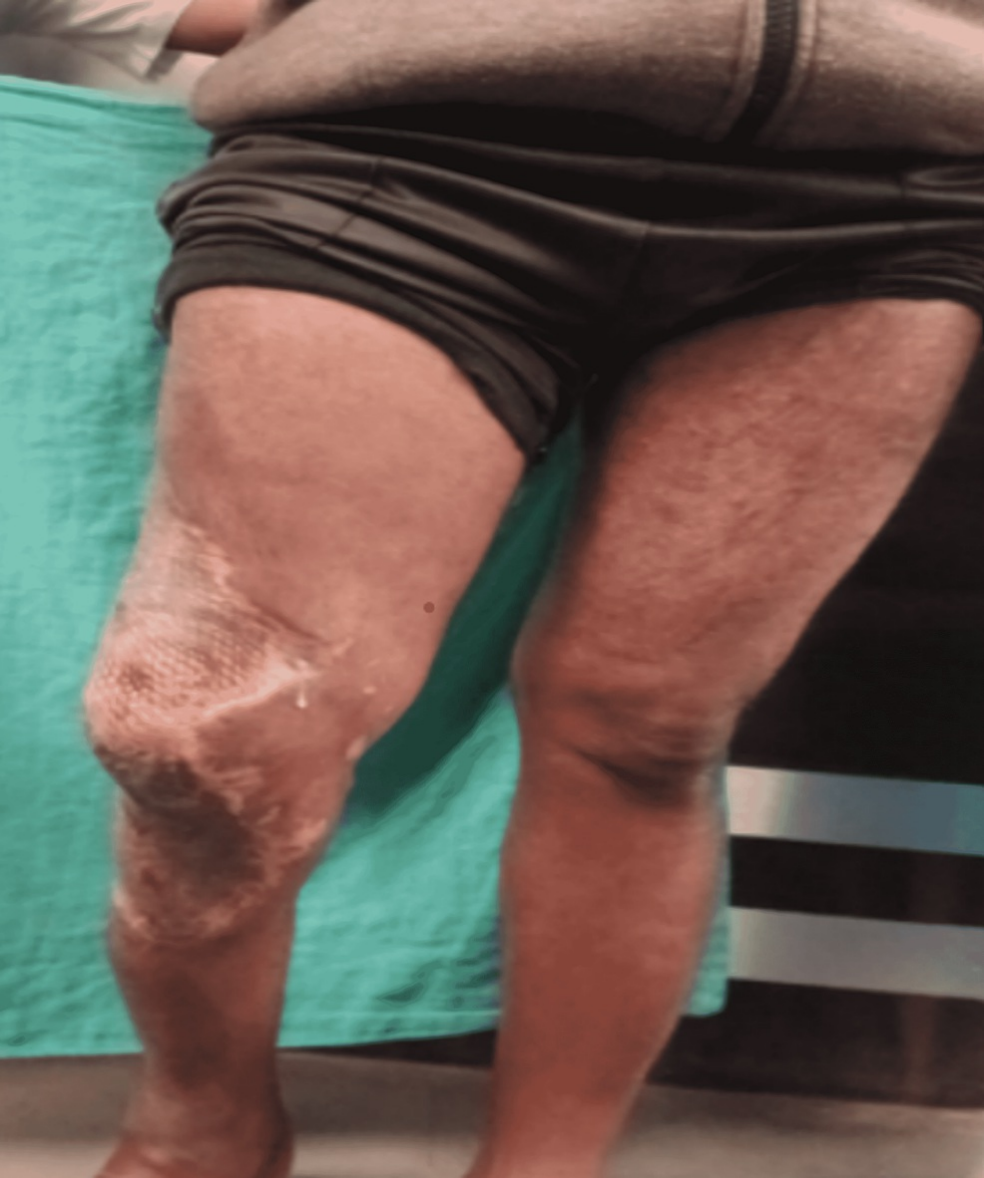
Healed lesion with good clinical outcome.

All the necrosed tissue was removed and a thorough wash was performed with a mixture of normal saline, betadine, and hydrogen peroxide. Sterile dressing was done. The patient was started on colistin as the culture showed growth of *Escherichia coli* sensitive only to colistin. The patient was managed with daily dressing, repeat debridement was done after four days of the first procedure, and vacuum-assisted closure (VAC) was done. The wound was found to be healthy after five days (Figure 3) and was further taken for split-thickness skin grafting and healed well. Follow-up at six months showed a united fracture in the femur (Figure 4) and a properly healed graft site (Figure 5).

## Discussion

The MLL is a sequel of closed degloving injury which involves the separation of the skin and subcutaneous fat from the underlying fascia. This results in the development of potential space between the two layers. This space gets filled with blood, fat, and lymphatic fluid. Often, this fluid gets colonized with a bacterial infection which leads to perioperative infection. In some cases, the granulation tissue that gets collected around the lesion develops into a pseudocapsule and the collected fluid is unable to get reabsorbed which leads to the development of a chronic fluid collection at the local site. This lesion was first described in the hip region. Other common locations for this condition are the pelvis, lower back, buttocks, and the lateral thigh region.

In some cases, the lesion is asymptomatic but, in most cases, it is associated with pain and swelling. The patient may or may not recall a history of trauma. Often, the lesion is misdiagnosed as local edema due to trauma. The delay in diagnosis leads to the swelling becoming tense and extensive cellular destruction. Because this lesion is an uncommon occurrence, a localized swelling in a polytrauma patient is often thought to be due to contusion or local edema [3,4].

MLL is mainly a clinical diagnosis and imaging modalities should be used for confirmation. A localized painful swelling in an emergency setting in a polytrauma patient should raise the suspicion of MLL. An ultrasound can be performed initially that will demonstrate the lesion as an anechoic area. The modality of choice is MRI as it shows excellent soft-tissue construct. Doppler can be used to rule out vascular causes such as deep vein thrombosis. CT scan may also demonstrate fluid collection but is not routinely recommended.

This condition can be managed conservatively as well as surgically depending on various factors such as the volume of the cavity, chronicity of the lesion, and pain and discomfort experienced by the patient. In acute conditions, conservative management with compression, rest, and ice fomentation can be helpful. In chronic cases where the lesion is large and non-responsive to conservative management, needle aspiration and drainage should be performed. Needle aspiration should be considered where the fluid collection is less than 50 mL. Cases where the collection is more than 50 mL, the lesion usually recurs. Hence, the collection of more than 50 mL is an indication of operative intervention [5]. Operative management includes simple incision and drainage. The incision is taken over the most prominent part of the swelling, fluid is drained, and the cavity is irrigated with normal saline and betadine. The lesion may be kept open or closed with VAC application. Recently, sclerosing agents such as doxycycline have been used for the obliteration of the cavity. Sclerosing agents cause destruction of the cells in the periphery of the lesion and induce fibrosis, thus causing obliteration of the cavity.

## Conclusions

Although MLLs are uncommon, doctors should be wary of patients who report chronic pain and fluid collection on advanced imaging following blunt trauma injuries. If identified quickly, conservative therapy can be undertaken; however, if left untreated, surgical intervention may be required, as detailed in this case. This case emphasizes the need of maintaining a high clinical suspicion for an MLL in cases of extensive closed soft-tissue injuries with or without fractures. A thorough follow-up and step-wise treatment help in the early detection, treatment, and prevention of rare but potentially fatal sequelae.

## References

[1] Morel-Lavallée VA (1863). [Decollements traumatiques de la peau et des couches sous-jacentes]. Arch Gen Med.

[2] Scolaro JA, Chao T, Zamorano DP (2016). The Morel-Lavallée lesion: diagnosis and management. J Am Acad Orthop Surg.

[3] Hak DJ, Olson SA, Matta JM (1997). Diagnosis and management of closed internal degloving injuries associated with pelvic and acetabular fractures: the Morel-Lavallée lesion. J Trauma.

[4] Nair AV, Nazar P, Sekhar R, Ramachandran P, Moorthy S (2014). Morel-Lavallée lesion: a closed degloving injury that requires real attention. Indian J Radiol Imaging.

[5] Pikkel YY, Hasan MJ, Ben-Yehuda Raz D, Ben Naftali Y, Duek OS, Ullman Y (2020). Morel Lavallée lesion - a case report and review of literature. Int J Surg Case Rep.

